# Effect of Photoanode Modification with Rare-Earth Metal Oxides on DSSC Performance

**DOI:** 10.3390/ma19142949

**Published:** 2026-07-09

**Authors:** Paweł Gnida, Natalia Żak, Anna Gawron, Marcin Libera, Wojciech A. Pisarski, Joanna Pisarska, Ewa Schab-Balcerzak

**Affiliations:** 1 Centre of Polymer and Carbon Materials, Polish Academy of Sciences, 34 M. Curie-Sklodowska Street, 41-819 Zabrze, Poland; agawron@cmpw-pan.pl; 2 Institute of Chemistry, Faculty of Science and Technology, University of Silesia, 9 Szkolna Street, 40-006 Katowice, Poland; natalia.zak@us.edu.pl (N.Ż.); marcin.libera@us.edu.pl (M.L.); wojciech.pisarski@us.edu.pl (W.A.P.); joanna.pisarska@us.edu.pl (J.P.); 3 SPIN-Lab Centre for Microscopic Research on Matter, University of Silesia in Katowice, 75 Pułku Piechoty 1A, 41-500 Chorzów, Poland

**Keywords:** dye-sensitized solar cells, DSSCs, titanium dioxide, TiO_2_, rare-earth elements, reduced light intensity

## Abstract

Modification of DSSC photoanodes by adding various types of materials is a widely used method to improve the performance of electrochemical devices. In this work, selected rare-earth oxide (RE_2_O_3_) nanoparticles, such as erbium oxide (Er_2_O_3_), holmium oxide (Ho_2_O_3_), neodymium oxide (Nd_2_O_3_), and ytterbium oxide (Yb_2_O_3_), were applied for photoanode preparation. A simple method for photoanode fabrication based on the direct mixing of oxide nanoparticles with TiO_2_ paste was employed. The effect of variation in the rare-earth oxide content in the photoanode was investigated. The prepared composite photoanodes were characterized by XRD and FE-SEM for structural and morphological studies. XRD verified the anatase polymorph of TiO_2_; FE-SEM with EDS mapping validated the uniform morphology of the anode and the distribution of RE_2_O_3_, as well as its amount. The photovoltaic parameters of modified DSSCs based on the current-voltage measurements were analyzed.

## 1. Introduction

Dye-sensitized solar cell (DSSC) technology is a highly promising approach for renewable energy generation, particularly in applications where conventional silicon-based photovoltaic (PV) systems are impractical. This is due to DSSCs’ advantages, including simple manufacturing processes that do not require high temperatures, vacuum environments, and high-cleanroom conditions, as well as the ability to operate efficiently over a wide range of light incidence angles and under low radiation intensity, as well as semi-transparency and tunable color [1]. Key trends in the DSSC market include their growing use in building-integrated photovoltaics (BIPV), where they are incorporated into windows, façades, and skylights, as well as their application in indoor, shaded, and off-grid environments, including integration into consumer electronics, wearable devices, IoT, and sensors [2,3]. Currently, the photoconversion efficiency of DSSCs has reached 15.2% [4].

DSSCs consist of a photoelectrode comprising an FTO coating with an inorganic semiconductor with adsorbed dye molecules, an electrolyte, and a counter electrode, all of which are critical to the performance and stability of the device. However, the photoanode plays a crucial role in the photon-to-electron conversion process by providing covalent anchoring of dye molecules and facilitating the transport of photoinduced electrons from the dye to the external circuit. In turn, an essential component of the photoelectrode, in addition to the dye, is a wide-band-gap metal oxide semiconductor [1,5]. It was found that, among the various investigated metal oxide semiconductors, titanium dioxide (TiO_2_) is the most suitable for DSSC applications [6,7]. However, it is not without drawbacks, including inherent poor light absorption ability, trap states and extensive electron-hole recombination, and has inferior charge mobility that limit cell efficiency; therefore, various modifications of the TiO_2_ electrode have been investigated [5,7]. Three main approaches to improve the optical, electronic, and chemical features of photoanodes with TiO_2_ can be distinguished: surface modification by coating TiO_2_ with a thin layer of an insulator or another semiconductor, introducing metal or non-metal additives into the TiO_2_ structure, and morphological variation in TiO_2_ nanocrystals (e.g., changes in size and shape) to improve the photoelectrical properties of DSSCs [1,5,6,7,8,9,10,11].

Among various materials, those based on rare-earth ions (RE^3+^) are also used to modify the photoanode designed for indoor applications of DSSCs [11,12]. According to the literature, such an approach typically improves device performance; however, the type of used compounds with RE^3+^ and its percentage contribution are crucial factors [13,14,15]. Compounds with RE^3+^ can be incorporated into the photoanode as an external layer or RE^3+^ ions can themselves act as modifiers [8,11,14,16,17]. It was found that deposition of RE^3+^-containing external layers may act as an energy barrier that prevents electron back-transfer or as a light-scattering layer, and it also influences the excited electron transportation process and the dye adsorption ability [9]. RE^3+^-modified nanoparticles can be utilized as spectral converters which help capture near-infrared and ultraviolet light radiation by converting it into visible wavelengths absorbable by the dye, and they can also be incorporated into DSSCs as a separate converting layer [8,16]. The incorporation of RE ions into the TiO_2_ facilitates shallow trapping, extends charge carrier lifetimes, and enhances charge transport. Moreover, modification of a TiO_2_ structure with RE^3+^-based compounds to increase the surface area resulted in higher adsorption of the dye [6,14]. Modification of TiO_2_ may positively influence the bandgap structure and the structural properties of this material, thereby enhancing the coupling between the TiO_2_ conduction band (CB) and the dye LUMO level [7]. Various preparative techniques of RE^3+^-modified TiO_2_ have been explored, including sol–gel, hydrothermal, vapor deposition, ball milling, and co-precipitation methods [8,18]. However, it was found that the addition of erbium oxide (Er_2_O_3_) nanoparticles to TiO_2_ via a simple method based on directly mixing erbium oxide nanoparticles with TiO_2_ paste resulted in a 81% increase in the photovoltaic efficiency of DSSCs. The percentage content of Er_2_O_3_ in composite photoanodes significantly affected the overall PV performance of the devices. The highest power conversion efficiency (PCE) was achieved in the DSSC with a composite photoelectrode prepared from a TiO_2_ semiconducting paste containing 1 wt.% Er_2_O_3_ nanoparticles. Such an enhancement in PCE was attributed to reduced charge recombination and improvement the optical performance of the composite photoanode, which enhanced open-circuit voltage (V_oc_) and short-circuit current density (J_sc_) due to the presence of an optimal amount of erbium oxide [14]. Incorporation of another oxide compound, Nb_2_O_5_, into a composite TiO_2_ photoelectrode leads to an increase in J_sc_, and consequently, enhanced power output [19].

Motivated by these findings, herein, we present our systematic investigations into the influence of different RE_2_O_3_ nanoparticles and their content in composite electrodes on the photovoltaic performance of DSSCs. In this study, the commercial ruthenium(II) bipyridyl complex N719 was used as the dye. Owing to its strong visible-light absorption, high molar extinction coefficient, excellent stability, and efficient electron transfer to the TiO_2_ conduction band, it is one of the most widely used dyes in current DSSC research, enabling high power conversion efficiencies [7,8].

## 2. Experimental Section

### 2.1. Materials

Fluorine-doped tin-oxide-coated glass slides (FTOs, 7 Ω/sq, Sigma-Aldrich, Merck, Steinheim, Germany), 18NR-T titania paste, PT1 Platinum Paste (Greatcell Solar Materials, Queanbeyan, NSW, Australia), a surfactant (Hellmanex III, Hellma Analytics, Müllheim im Markgräflerland, Germany), 2-propanol (IPA), tetrahydrofuran (THF), and titanium(IV) tetrachloride, di-tetrabutylammonium *cis*-bis(isothiocyanato)bis(2,2′-bipyridyl-4,4′-dicarboxylato)ruthenium(II) (N719); erbium oxide, >99.99%; and EL-HSE electrolyte (I_3_¯/I¯, solvent: 3-methoxypropionitrile) were purchased from Sigma-Aldrich (Merck, Steinheim, Germany). For photoanode preparation, the neodymium oxide, 99.99% (Thermo Scientific, Waltham, MA, USA); holmium oxide, 99.99%; and ytterbium oxide, 99.99% (Chempur, Karlsruhe, Germany) were used. *tert*-Butyl alcohol (*t*-BuOH) (Chempur, Piekary Śląskie, Poland) and acetonitrile (Sigma-Aldrich, Merck, Steinheim, Germany) were applied in device preparation.

### 2.2. Synthesis of Composite for Photoanode Preparation

In the present procedure, the TiO_2_ paste modified by adding 0.5, 1, 2, and 3 wt.% Er_2_O_3_, Yb_2_O_3_, Ho_2_O_3_ or Nd_2_O_3_ was obtained. The appropriate amounts of individual components, i.e., TiO_2_ paste and Er_2_O_3_, Yb_2_O_3_, Ho_2_O_3_, or Nd_2_O_3_ were introduced into an agate mortar and mixed with a pestle for 1 h to ensure sufficient samples’ homogeneity.

### 2.3. Fabrication of DSSCs

Initially, the glass substrates coated with an approximately 500 nm-thick fluorine-doped tin oxide layer were subjected to a multistep cleaning procedure. The substrates were first immersed in a 10% aqueous surfactant solution (Hellmanex, Müllheim im Markgräflerland, Germany) and treated in an ultrasonic bath for 15 min at 40 °C. Subsequently, they were cleaned with distilled water and sonicated again under identical conditions; this rinsing procedure was repeated twice to ensure effective removal of contaminants. Following the distilled water cleaning steps, the substrates were immersed in isopropanol (IPA) and sonicated for 15 min at 40 °C. After being removed from the IPA and allowed to dry thoroughly under ambient conditions, three layers of TiO_2_ paste (18NR-T, Greatcell Solar Materials, Queanbeyan, NSW, Australia) modified with RE_2_O_3_ oxides were deposited onto the FTO substrates using a screen-printing method. Each printed layer was preheated at 125 °C for 5 min to achieve the desired film thickness and stability. Upon deposition of the final layer, the films were sintered at 500 °C for 30 min. Finally, the TiO_2_-coated FTO substrates were allowed to cool to room temperature. The active geometric area of the screen-printed TiO_2_ layer was fixed at 0.64 cm^2^ (0.80 cm × 0.80 cm), and the measurements were performed directly on this area without an external aperture mask. Before immersion, the photoanodes were preheated to 80 °C. The photoanodes were prepared by immersing the anodes in N719 solutions at a concentration of 3 × 10^−4^ M in ACN:t-BuOH (1:1, *v*:*v*) for 24 h. The counter electrode was fabricated on FTO glass substrates that were cleaned using the same protocol as for TiO_2_ deposition. A commercial paste containing platinum nanoparticles was applied via screen printing to form a single uniform layer. The printed films were subsequently sintered in a muffle furnace at 450 °C for 30 min under an ambient atmosphere. The DSSC was fabricated by assembling the prepared photoanode and the counter electrode, with the inter-electrode gap filled with a commercial liquid electrolyte (EL-HSE) containing the I^−^/I_3_^−^ redox couple. The platinum-based counter electrodes were fabricated using a standardized screen-printing technique. Three layers of platinum paste were deposited onto the pre-cleaned FTO glass substrates. Following the printing process, the electrodes were subjected to thermal treatment at 450 °C for 30 min in air to form a uniform platinum layer.

The blocking layers (BLs) were prepared according to the following procedure. A 2 mol·dm^−3^ stock solution was prepared from commercial titanium(IV) tetrachloride (TiCl_4_). Pre-cleaned FTO substrates were placed in a Petri dish containing 48.75 mL of deionized water, followed by the addition of 1.25 mL of the 2 mol·dm^−3^ TiCl_4_ solution to obtain a final concentration of 0.05 mol·dm^−3^. The resulting solution was maintained at 70 °C for 30 min. After treatment, the substrates were removed, gently rinsed with distilled water, and subsequently annealed in a furnace at 500 °C for 30 min.

The TiCl_4_ post-treatment (TiCl_4_ P-T) was performed on the active layers, i.e., FTO/TiO_2_ substrates. The procedure for this chemical post-treatment was identical to that used for the blocking layer; specifically, the prepared anodes were immersed in a TiCl_4_ precursor solution. The system was heated at 70 °C for 30 min, after which the substrates were removed, rinsed, and annealed at 500 °C.

The solar cells were assembled in a simplified open-faced sandwich configuration held together firmly using customized mechanical clips, without additional polymer spacers or thermal-sealing protocols. To ensure reproducibility, each type of solar cell was fabricated and measured in triplicate (three independent devices), and the average values have been reported. The liquid electrolyte was introduced into the active junction area via direct capillary injection between the two clamped electrodes. Prior to the measurements, the solar simulator was fully calibrated under AM 1.5G solar illumination 100 mW cm^−2^ using a standard silicon reference cell certified by the National Renewable Energy Laboratory (NREL). The J–V characteristics were recorded using a digital source meter within a voltage range of 2.0 V and a current range of 100 mA. Data were collected using a continuous forward-to-reverse (F → R) scan direction with a voltage step, scan rate 1000 mV s^−1^ ΔV_out_ of 10 mV. The forward and reverse limits were strictly set to 1.0 V and 0.05 A, with an instrument delay time maintained before data acquisition at each voltage step. All measurements were conducted at a controlled ambient temperature of 25 °C.

#### Dye-Loading Analysis

The N719 dye was desorbed from the TiO_2_ surface using a 0.01 M aqueous NaOH solution. A calibration curve was prepared for the dye solutions in the concentration range of 8·10^−6^ to 1·10^−4^ M, and their UV–Vis absorption spectra were recorded. The desorption of the dye from the photoanodes was performed by immersing them in the solution for 1 h until the dye was completely removed. Complete desorption of the dye was confirmed by UV–Vis absorption measurements of the TiO_2_ substrates after their removal from the solution. UV–Vis absorption spectra were recorded for the obtained solutions, and the concentration was determined based on the calibration curve. Subsequently, the concentration of the solution was converted to moles. Taking into account the actual active area of the photoanode, the resulting number of moles was calculated per 1 cm^2^.

### 2.4. Characterization

Current–voltage (J–V) characteristics were recorded using a Keithley 2400 SourceMeter (Tektronix, Inc., Beaverton, OR, USA) operated with dedicated software and a PV Solutions solar simulator delivering a standard irradiance of 100 mW·cm^−2^ under AM 1.5G conditions (or, for selected devices, under illumination intensities ranging from 10 to 90 mW·cm^−2^). UV–Vis spectra were acquired using a Jasco V-750 UV–Vis–NIR spectrophotometer (Jasco Inc., Tokyo, Japan). Phase analysis of the samples was performed using a Bruker D8 Advance diffractometer (Bruker Corporation, Billerica, MA, USA) equipped with a copper X-ray source (λ = 1.54 Å), operating at 40 kV and 40 mA, and a LYNXEYE-XT detector. Measurements were carried out in θ–2θ scanning mode over an angular range of 10–90° 2θ, with a step size of 0.02° and a scanning rate of 0.3°/min. The measurements were carried out at four points (one at the TiO_2_/metal and FTO interfaces, and three on the TiO_2_ layer containing rare-earth metals) using a Tescan Amber X scanning electron microscope (Tescan, Brno, Czech Republic). The EDS analysis was performed at 20 kV and 3 nA using an Ultim Max 100 detector (Oxford Instruments, High Wycombe, UK).

## 3. Results and Discussion

The effect of both the type (Er_2_O_3_, Ho_2_O_3_, Nd_2_O_3_, and Yb_2_O_3_) and amount (0.5, 1, 2, and 3 wt.%) of rare-earth oxides in the photoanode on DSSC performance was evaluated. In the first step of investigation, the prepared anodes as TiO_2_ layers with 0.5 wt.% of rare-earth oxides deposited on FTO were structurally characterized by X-ray diffraction (XRD) measurements. Next, the distribution of RE_2_O_3_ on the TiO_2_ layer was determined using Field Emission Scanning Electron Microscopy–Energy-Dispersive X-ray Spectroscopy (FE-SEM-EDS). The DSSCs were constructed using the commercial N719 dye as a sensitizer and electrolyte-bearing iodide redox couple (EL-HSE). Additionally, for selected devices, their structure was modified by the introduction of blocking (BL) and TiCl_4_ post-treatment. The photovoltaic parameters of open-circuit voltage, short-circuit current density, fill factor (FF), and power conversion (PCE) were calculated from current-voltage characteristics registered under standard − 100 mW cm^−2^ and reduced to 10 mW cm^−2^ illuminations.

### 3.1. XRD Characterization

**Figure 1:**
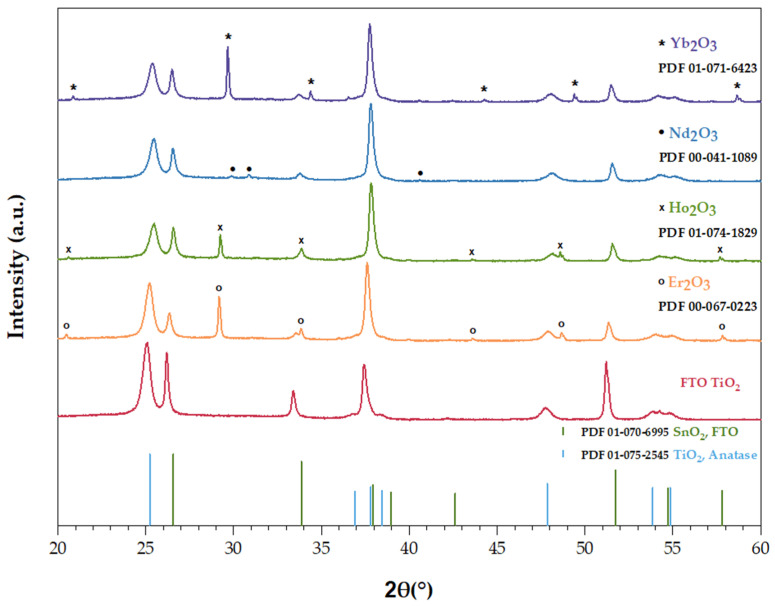
XRD patterns of TiO_2_ layers modified with Er_2_O_3_, Ho_2_O_3_, Nd_2_O_3_, and Yb_2_O_3_ deposited on FTO.

The FTO/TiO_2_:RE_2_O_3_ anodes were mounted on dedicated standard holders for solid-state materials, including thin films. The obtained diffraction patterns were analyzed using DIFFRAC.EVA software and compared with reference crystallographic data from the PDF-4 ICDD (International Centre for Diffraction Data) and COD (Crystallography Open Database) databases. Crystalline phases were identified based on the positions and relative intensities of the diffraction peaks. Figure 1 presents XRD patterns of TiO_2_ layers modified with Er_2_O_3_, Ho_2_O_3_, Nd_2_O_3_, and Yb_2_O_3_ deposited on fluorine-doped tin oxide conductive glass substrates.

The diffraction pattern of the Er_2_O_3_-containing sample at 29.2°, 33.9°, and 48.6°, corresponding to the [222], [400], and [440] planes (Space Group: Ia-3 (206)), according to PDF card 00-067-0223, indicates the presence of the cubic erbium oxide phase. For the Ho_2_O_3_ sample, reflections located at 29.1°, 33.6°, and 48.5°, assigned to the [222], [400], and [440] planes (Space Group: Ia-3 (206)), confirm the occurrence of the cubic holmium oxide structure (PDF 00-063-0304). Diffraction maxima at 29.7°, 30.7°, and 40.5° for the Nd_2_O_3_ sample were assigned to the [002], [101], and [102] planes, respectively, characteristic of the hexagonal neodymium oxide phase belonging to Space Group: P-3m1 (164), (PDF 00-041-1089). In the case of the Yb_2_O_3_ sample, diffraction peaks located at 29.6°, 34.3°, and 49.4°, corresponding to the [222], [400], and [440] planes, indicate the presence of the cubic ytterbium oxide phase, Space Group: Ia-3 (206), in agreement with PDF card 01-071-6423. The observed diffraction reflections confirm the successful dispersion of rare-earth oxides within the investigated thin-film systems. In addition, reflections observed for the FTO TiO_2_ sample can be assigned to two tetragonal crystalline structures corresponding to titanium oxide and tin oxide. The TiO_2_ phase was identified based on diffraction peaks located at 25.2°, 37.8°, and 47.8°, corresponding to the [101], [004], and [200] crystallographic planes, respectively (PDF 01-070-7348, Space Group: I41/amd (141)). These results indicate the presence of anatase, a metastable polymorphic phase of TiO_2_. Diffraction maxima located at 2θ values of 26.6°, 33.9°, and 51.7°, corresponding to the [110], [101], and [211] planes, indicate the presence of SnO_2_ with a tetragonal structure belonging to Space Group: P42/mnm (136), according to PDF card 01-070-6995. The diffraction patterns of the remaining samples also correspond to the structural characteristics of the SnO_2_ substrate and TiO_2_ in the anatase phase. The pristine TiO_2_ on the FTO sample was used as a structural reference for comparison with the rare-earth-modified TiO_2_ coatings.

The obtained XRD results are consistent with other characterization studies performed to confirm the presence of both anatase TiO_2_ and appropriate RE_2_O_3_ phase (where RE = Er, Ho, Nd, or Yb).

### 3.2. FE-SEM EDS

**Figure 2:**
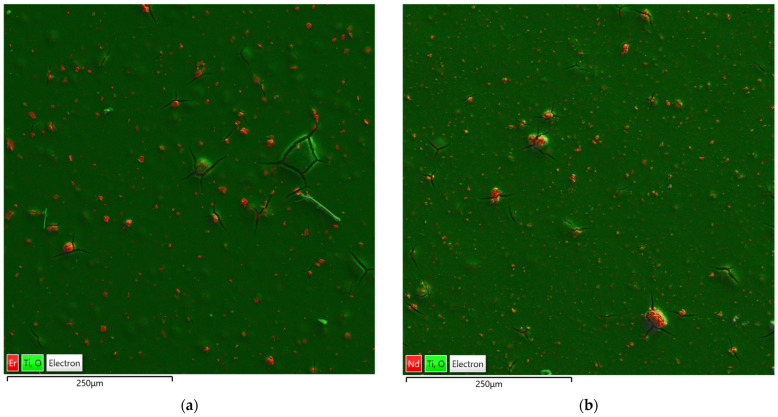

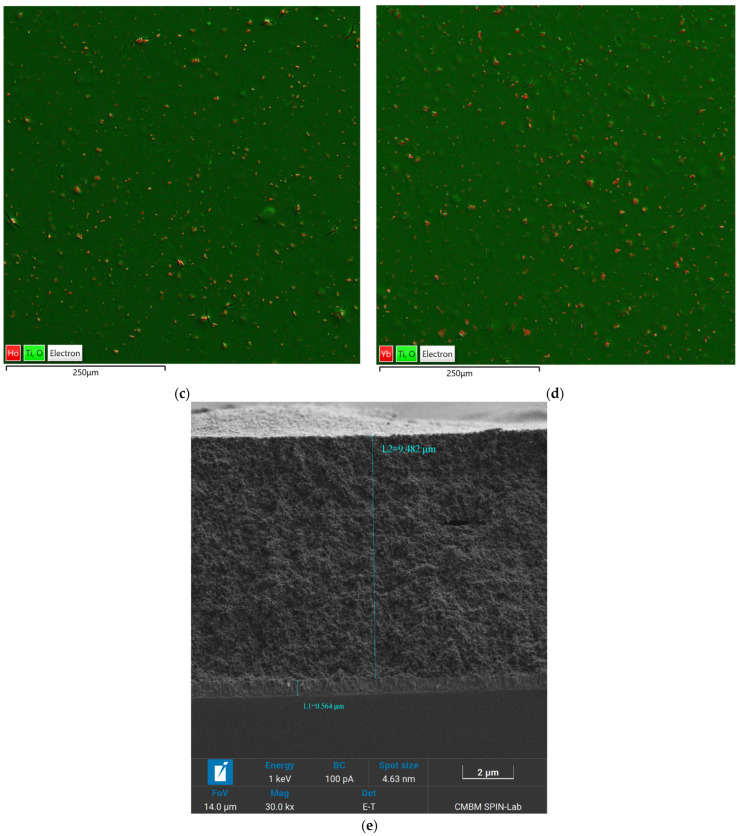
Representative FE-SEM EDS surface images of the TiO_2_ photoanodes modified with a 2 wt.% addition of (**a**) Er_2_O_3_, (**b**) Nd_2_O_3_, (**c**) Ho_2_O_3_, (**d**) Yb_2_O_3_, and (**e**) cross-section of anode Glass/FTO/BL/TiO_2_:Yb_2_O_3_/TiCl_4_ P-T.

FE-SEM EDS measurements were conducted to verify the dispersion of rare-earth oxides on the TiO_2_ layer. To ensure the homogeneity of the modifier distribution, three distinct areas were imaged for each sample. Figure 2 displays typical surface micrographs of the TiO_2_ anodes containing 2 wt.% of the respective oxide additives.

Regarding the microstructural features of the prepared photoanodes, a uniform macro-distribution of the rare-earth oxide additives within the TiO_2_ layer was successfully confirmed across all investigated weight percentages. However, distinct differences in the layer morphology and crack formation were observed depending on the specific type of the introduced modifier. In the case of the Er_2_O_3_ and Nd_2_O_3_ additives, the largest number of surface aggregates was observed, which may adversely affect charge transport efficiency and restrict the maximum amount of dye anchoring, potentially reducing in the overall photovoltaic performance. Furthermore, structural damage to the TiO_2_ layer was observed in the regions with the largest aggregates, which could significantly degrade the interfacial contact within the photoanode, and consequently, lower the PCE of the device [20]. In contrast, the films containing Ho_2_O_3_ exhibited a reduced number of microcracks compared to the Er_2_O_3_ and Nd_2_O_3_ systems. Among all samples, the Yb_2_O_3_-bearing layers consistently exhibited superior structural quality, showing the highest resistance to crack formation in every single case. Furthermore, the agglomerates formed in the matrices containing Ho_2_O_3_ and Yb_2_O_3_ maintained significantly smaller dimensions than those observed in the films modified with Er_2_O_3_ and Nd_2_O_3_, regardless of the oxide content.

### 3.3. Photovoltaic Performance

**Figure 3:**
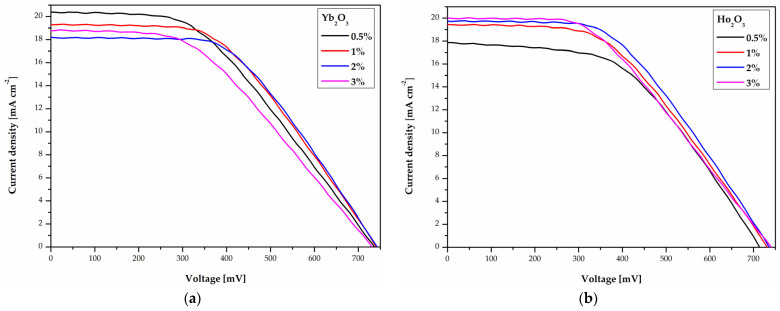
Current density–voltage characteristics of solar cells with photoanodes containing (**a**) Yb_2_O_3_ and (**b**) Ho_2_O_3_.

To investigate the influence of rare-earth oxides and their concentrations, solar cells were fabricated and systematically analyzed. Specific amounts of individual oxides were added into the TiO_2_ paste at concentrations of 0.5, 1, 2, and 3 wt.%. However, it should be noted that the weight percentages of rare-earth oxides were determined relative to the paste containing TiO_2_ nanoparticles, rather than the mass of TiO_2_ after firing, which accounts for the previously described differences. To ensure clarity, the initial, nominal weight percentages of the oxide relative to the paste will be used throughout the remainder of this study. Initial efforts focused on screening both the oxide type and its optimal content. For this purpose, a standard cell configuration was utilized, specifically structured as glass/FTO/TiO_2_:RE_2_O_3_@N719/EL-HSE/Pt/FTO/glass (where RE = Er, Nd, Yb, or Ho). To ensure statistical reliability, three identical devices were fabricated for each cell type, allowing for the determination of average photovoltaic parameters. The photovoltaic parameters, including open-circuit voltage (V_oc_), short-circuit current density, fill factor, and power conversion efficiency (PCE), were derived from the recorded current density–voltage (J–V) curves (Figure 3) and are summarized in Table 1.

**Figure 4:**
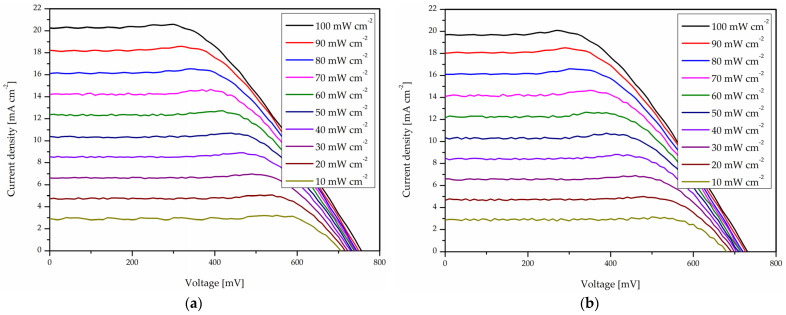
The J–V characteristics of solar cells based on photoanode structures of (**a**) glass/FTO/BL/TiO_2_:Ho_2_O_3_ @N719/TiCl_4_ P-T and (**b**) glass/FTO/BL/TiO_2_:Yb_2_O_3_@N719/TiCl_4_ P-T.

Alongside the modified solar cells, a reference device with the configuration glass/FTO/TiO_2_@N719/EL-HSE/Pt/FTO/glass was fabricated, exhibiting an average power conversion efficiency (PCE) of 6.29%. Comparison with the modified devices demonstrated that the photovoltaic performance strongly depended on both the type and concentration of the added rare-earth oxide. For the Er_2_O_3_-modified cells, the highest efficiency (6.28%) was achieved at an additive loading of 1%, which was comparable to that of the reference device. In contrast, the remaining investigated concentrations (0.5%, 2%, and 3%) resulted in lower PCE values of 5.90%, 5.33%, and 5.27%, respectively. A similar trend was observed for Nd_2_O_3_; although the optimum loading was also observed at 1%, the maximum efficiency achieved (6.18%) remained slightly lower than that of the reference cell. These results suggest that the addition of erbium(III) oxide or neodymium(III) oxide does not improve DSSC performance under the investigated experimental conditions. Consequently, these materials were excluded from further studies. In contrast, the incorporation of both Yb_2_O_3_ and Ho_2_O_3_ resulted in a consistent enhancement of conversion efficiency across all investigated concentrations. In both cases, the optimal additive concentration was found to be 2 wt.%, yielding average PCE values of 6.95% and 7.03% for the Yb_2_O_3_- and Ho_2_O_3_-modified devices, respectively. In all cases, the addition of RE_2_O_3_ affected the open-circuit voltage of the devices. For the cells modified with Er_2_O_3_, a slight decrease in V_oc_ was observed. In contrast, the remaining three additives produced a more pronounced effect, namely an increase in V_oc_. This enhancement may be associated with shifts in the TiO_2_ electron Fermi level and changes in charge carrier density within the photoanode [21]. The reduced J_sc_ values observed for the Er_2_O_3_- and Nd_2_O_3_-containing devices could be attributed to the formation of larger aggregates within the TiO_2_ layer, as well as the presence of pronounced surface cracks that hindered charge transport. Since only the incorporation of Yb_2_O_3_ and Ho_2_O_3_ resulted in improved photovoltaic performance, these modifiers were selected for further investigation. Moreover, for both oxides, the best results were obtained at a concentration of 2 wt.%, and therefore, subsequent studies were limited to this composition. Further investigations focused on enhancing device efficiency through the additional application of the blocking layer (BL) and TiCl_4_ post-treatment. In DSSCs, the blocking layer is primarily used to suppress charge recombination at the interface between the conductive substrate and the electrolyte, thereby reducing interfacial electron losses and increasing V_oc_ [22]. In contrast, this chemical TiCl_4_ post-treatment aims to optimize electron transport and decrease back-electron transfer to the electrolyte by passivating surface defects. Current–voltage measurements were subsequently carried out at reduced-light-intensity conditions in order to evaluate the potential application of the developed devices under attenuated AM 1.5G illumination. To determine the amount of anchored dye molecules on the modified substrates containing a 2 wt.% addition of rare-earth oxides, desorption studies were performed using a NaOH solution. Based on the measurements and a prepared calibration curve, the following amounts of dye molecules per 1 cm^2^ of active area were determined: 0.763 ∙ 10^−7^ mol cm^−2^ (Yb_2_O_3_), 0.863 ∙ 10^−7^ mol cm^−2^ (Er_2_O_3_), 0.888 ∙ 10^−7^ mol cm^−2^ (Ho_2_O_3_), and 1.180 ∙ 10^−7^ mol cm^−2^ (Nd_2_O_3_). Notably, the highest number of anchored dye molecules was found for the substrate with Nd_2_O_3_, which unfortunately did not translate into the highest efficiency, or at least the highest J_sc_ value. This could be attributed to the presence of numerous cracks within the TiO_2_ layer. For the remaining oxides, smaller differences in the number of anchored molecules were observed. Among them, the highest amount of dye molecules was anchored to the substrate with Ho_2_O_3_, which in this case correlated with a high J_sc_ value (19.75 mA cm^−2^). This suggests that while defective morphologies with microcracks can physically trap a large volume of dye, they simultaneously induce severe charge recombination and disrupt electronic continuity, whereas a uniform layer structure ensures effective charge injection. The illumination intensity was gradually decreased from the standard 100 mW cm^−2^ to 10 mW cm^−2^ in steps of 10 mW cm^−2^. For each device configuration, three nominally identical solar cells were fabricated to verify the reproducibility of the obtained results. Following reproducibility verification, one representative cell from each series was selected for detailed characterization under reduced illumination conditions. The recorded J–V curves are presented in Figure 4, while the corresponding photovoltaic parameters are summarized in Table 2.

Compared with the devices fabricated without a blocking layer and TiCl_4_ post-treatment, the modified solar cells—measured under standard illumination conditions (100 mW cm^−2^)—exhibited enhanced photovoltaic performance. In the case of Ho_2_O_3_-modified devices, the open-circuit voltage increased by approximately 20 mV, whereas a smaller increase of 4 mV was observed for Yb_2_O_3_-based cells. A similar trend was noted for the short-circuit current density, which reached values of up to 1.50 mA cm^−2^. The observed enhancement in photovoltaic parameters is likely attributable to reduced charge recombination resulting from the presence of the blocking layer, along with improved electron transport and enhanced charge collection efficiency enabled by the TiCl_4_ post-treatment. Consequently, the power conversion efficiency increased to 7.45% for the Ho_2_O_3_-modified cells and 7.20% for the Yb_2_O_3_-based devices.

**Figure 5:**
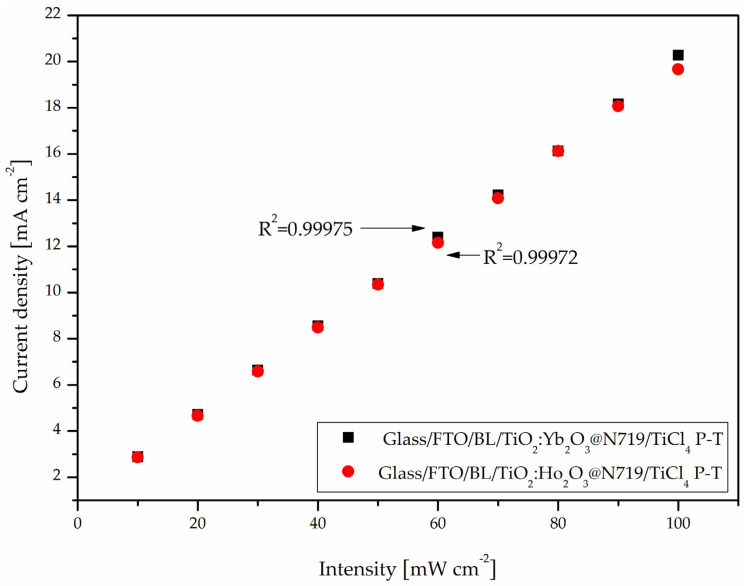
Dependence of the short-circuit current density J_sc_ on light intensity for the investigated solar cells.

With decreasing light intensity, a decrease in open-circuit voltage was observed from 756 to 706 mV (a 50 mV drop) for the Ho_2_O_3_-modified device, and from 739 to 681 mV (a 58 mV drop) for the Yb_2_O_3_-based solar cell. The light-intensity dependence of V_oc_ is fundamentally governed by the interplay between charge generation and carrier lifetime within the device. Under high illumination, accelerated charge generation dominates over carrier lifetime reduction, increasing V_oc_ [23]. Conversely, as the incident light intensity decreases, carrier lifetime limits performance more significantly than the generation rate, resulting in the observed voltage decline. Regarding J_sc_, a continuous decrease in values was observed with each reduction in the incident light intensity. Specifically, for the Ho_2_O_3_-modfied device, a drop from 20.28 to 2.89 mA cm^−2^ (a decrease of 17.39 mA cm^−2^) was recorded when lowering the intensity from 100 to 10 mW cm^−2^. An identical trend was evident for the Yb_2_O_3_-containing devices, which initially exhibited a J_sc_ of 19.67 mA cm^−2^ at 100 mW cm^−2^ and ultimately reached 2.87 mA cm^−2^ at 10 mW cm^−2^, marking a total decline of 16.80 mA cm^−2^. This behavior is primarily attributed to the reduced photon flux reaching the active layer under lower illumination levels, which inherently diminishes the charge carrier generation rate within the photoanode. Importantly, as illustrated in Figure 5, J_sc_ exhibits a strictly linear dependence on light intensity, confirmed by the exceptionally high coefficient of determination values (R^2^ = 0.99975 and 0.99972 for the Ho_2_O_3_- and Yb_2_O_3_-modified cells, respectively). This near-ideal linearity suggests that the charge collection process is maintained across the investigated range, potentially implying that severe transport bottlenecks or significant space-charge accumulation within the modified semiconductor matrix are minimized under these attenuated illumination levels. A significant increase in the fill factor was observed upon decreasing illumination intensity, rising from 0.49 to 0.90 and from 0.50 to 0.83 for the investigated devices. The increase in FF under lower illumination may be associated with reduced recombination [24,25,26]. Evaluating photovoltaic parameters under such reduced light intensities serves as a crucial benchmark for assessing the practical viability of DSSCs under low-light AM 1.5G conditions. Similar systematic investigations under controlled low-light conditions are increasingly reported in the recent literature; for instance, Lu et al. [27] focused on the stepwise optimization of dye-sensitized solar cells specifically operating under a dim indoor illumination of 50 lux.

In conclusion, these modifications of the photovoltaic parameters under reduced light intensity ultimately resulted in power conversion efficiencies of 18.11% for the Ho_2_O_3_-modified device and 16.09% for the Yb_2_O_3_-based solar cell at an illumination of 10 mW cm^−2^. This behavior is hypothesized to contribute to a favorable balance between carrier generation and reduced loss pathways [12]. Given the high performance maintained under weak illumination, these systems potentially hold promise for applications requiring efficient light harvesting under reduced irradiance and attenuated illumination conditions.

## 4. Summary

In this work, TiO_2_ pastes containing rare-earth oxide additives—Er_2_O_3_, Nd_2_O_3_, Ho_2_O_3_, and Yb_2_O_3_—were successfully prepared. The presence of these oxide additives within the TiO_2_ layers was confirmed via XRD analysis. FE-SEM micrographs revealed a uniform distribution of the nanoparticles; however, the dispersion of Er_2_O_3_ and Nd_2_O_3_ induced the formation of larger aggregates, which occasionally disrupted the film continuity and initiated microcracks. Photovoltaic parameters indicated that the addition of 2 wt.% of either Ho_2_O_3_ or Yb_2_O_3_ yielded the most favorable performance. Under standard illumination conditions, these devices exhibited PCEs of 7.03% and 6.95%, respectively. On the other hand, the introduction of Er_2_O_3_ and Nd_2_O_3_ resulted in either negligible effects or a distinct degradation of the device efficiency. The application of the aforementioned oxide additives resulted, in most cases, in an increase in V_oc_ by 8 to 23 mV, as well as an improvement in FF in the range of 0.02 to 0.10, which translated into the observed enhancement in device efficiency. Consequently, further investigations focused on implementing the blocking layer and the TiCl_4_ post-treatment into the optimized cell architectures containing Ho_2_O_3_ and Yb_2_O_3_. Under standard illumination conditions, these systems achieved efficiencies of 7.45% and 7.20%, respectively. Photovoltaic testing of fabricated DSSCs was conducted under reduced light intensity. At an illumination intensity of (10 mW cm^−2^), this achieved average power conversion efficiencies of 18.11% and 16.09% for the systems modified with Ho_2_O_3_ and Yb_2_O_3_, respectively. These promising efficiencies obtained under reduced light intensity indicate a strong potential for low-light photovoltaic applications, though further comprehensive investigations under actual indoor spectra are required.

## Figures and Tables

**Table 1:** Photovoltaic parameters of DSSCs sensitized with N719 under standard illumination (100 mW cm^−2^).

Rare-Earth Metal Oxide	Percentage Content [wt.%]	V_oc_ [mV]	J_sc_ [mA cm^−2^]	FF	PCE [%]
**-**	-	723 ± 10	20.19 ± 0.21	0.42 ± 0.02	6.29 ± 0.05
**Er_2_O_3_**	0.5	715 ± 13	15.04 ± 0.17	0.55 ± 0.01	5.90 ± 0.08
	1	720 ± 8	16.19 ± 0.22	0.55 ± 0.01	6.28 ± 0.03
	2	717 ± 11	17.04 ± 0.41	0.44 ± 0.02	5.33 ± 0.12
	3	692 ± 16	15.60 ± 0.14	0.49 ± 0.01	5.27 ± 0.09
**Nd_2_O_3_**	0.5	720 ± 7	16.66 ± 0.18	0.50 ± 0.02	5.99 ± 0.11
	1	746 ± 20	18.42 ± 0.38	0.45 ± 0.01	6.18 ± 0.07
	2	731 ± 13	5.28 ± 0.24	0.59 ± 0.01	2.30 ± 0.17
	3	673 ± 10	5.43 ± 0.29	0.52 ± 0.02	1.92 ± 0.09
**Yb_2_O_3_**	0.5	736 ± 8	20.38 ± 0.35	0.44 ± 0.01	6.61 ± 0.07
	1	741 ± 14	19.30 ± 0.26	0.49 ± 0.02	6.91 ± 0.12
	2	735 ± 21	18.16 ± 0.19	0.52 ± 0.01	6.95 ± 0.07
	3	731 ± 16	18.81 ± 0.34	0.44 ± 0.01	6.00 ± 0.08
**Ho_2_O_3_**	0.5	714 ± 8	17.87 ± 0.25	0.50 ± 0.01	6.33 ± 0.05
	1	732 ± 18	19.42 ± 0.38	0.47 ± 0.01	6.67 ± 0.12
	2	736 ± 9	19.75 ± 0.41	0.49 ± 0.01	7.03 ± 0.07
	3	740 ± 12	19.99 ± 0.46	0.46 ± 0.02	6.48 ± 0.16

**Table 2:** The photovoltaic parameters of modified DSSCs devices for a representative cell under reduced illuminations.

Photoanode	Intensity [mW cm^−2^]	V_oc_ [mV]	J_sc_ [mA cm^−2^]	FF	PCE [%]
Glass/FTO/BL/TiO_2_:Ho_2_O_3_@ N719/TiCl_4_ P-T	100	756	20.28	0.49	7.45
	90	749	18.16	0.53	8.02
	80	744	16.13	0.57	8.50
	70	741	14.23	0.60	8.97
	60	738	12.40	0.63	9.52
	50	733	10.39	0.67	10.18
	40	729	8.55	0.70	10.92
	30	723	6.63	0.75	12.01
	20	716	4.72	0.81	13.68
	10	706	2.89	0.90	18.11
Glass/FTO/BL/TiO_2_:Yb_2_O_3_@ N719/TiCl_4_ P-T	100	739	19.67	0.50	7.20
	90	726	18.07	0.52	7.56
	80	719	16.12	0.55	8.02
	70	716	14.09	0.59	8.47
	60	713	12.16	0.62	8.95
	50	709	10.35	0.65	9.50
	40	704	8.48	0.68	10.22
	30	699	6.58	0.73	11.19
	20	692	4.66	0.78	12.57
	10	681	2.87	0.83	16.09

## Data Availability

The original contributions presented in this study are included in the article. Further inquiries can be directed to the corresponding authors.
